# Comparative effectiveness of rituximab and cladribine in relapsing–remitting multiple sclerosis: A target trial emulation

**DOI:** 10.1177/13524585251342727

**Published:** 2025-05-26

**Authors:** Brit Ellen Rød, Einar A Høgestøl, Øivind Torkildsen, Kjetil Bjørnevik, Jon Michael Gran, Mathias H Øverås, Marton König, Kjell-Morten Myhr, Stig Wergeland, Gro O Nygaard

**Affiliations:** Neuro-SysMed, Department of Neurology, Haukeland University Hospital, Bergen, Norway; Department of Clinical Medicine, University of Bergen, Bergen, Norway; The Norwegian Multiple Sclerosis Registry and Biobank, Haukeland University Hospital, Bergen, Norway; Institute of Clinical Medicine, University of Oslo, Oslo, Norway; Department of Neurology, Oslo University Hospital, Oslo, Norway; Department of Psychology, Oslo University Hospital, Oslo, Norway; Neuro-SysMed, Department of Neurology, Haukeland University Hospital, Bergen, Norway; Department of Clinical Medicine, University of Bergen, Bergen, Norway; Departments of Epidemiology and Nutrition, Harvard T. H. Chan School of Public Health, Boston, MA, USA; Oslo Centre for Biostatistics and Epidemiology, Department of Biostatistics, Institute of Basic Medical Sciences, University of Oslo, Oslo, Norway; Institute of Clinical Medicine, University of Oslo, Oslo, Norway; Department of Neurology, Oslo University Hospital, Oslo, Norway; Institute of Clinical Medicine, University of Oslo, Oslo, Norway; Department of Neurology, Oslo University Hospital, Oslo, Norway; Neuro-SysMed, Department of Neurology, Haukeland University Hospital, Bergen, Norway; Department of Clinical Medicine, University of Bergen, Bergen, Norway; Neuro-SysMed, Department of Neurology, Haukeland University Hospital, Bergen, Norway; Department of Clinical Medicine, University of Bergen, Bergen, Norway; The Norwegian Multiple Sclerosis Registry and Biobank, Haukeland University Hospital, Bergen, Norway; Institute of Clinical Medicine, University of Oslo, Oslo, Norway; Department of Neurology, Oslo University Hospital, Oslo, Norway

**Keywords:** Relapsing–remitting multiple sclerosis, rituximab, B-cell depletion therapy, cladribine tablets, target trial emulation, efficacy, safety

## Abstract

**Background:**

Head-to-head comparisons of high-efficacy therapies for relapsing–remitting multiple sclerosis (RRMS) are lacking. We conducted a target trial emulation to compare the long-term effectiveness of rituximab and cladribine.

**Methods:**

We estimated the effect of initiating treatment with rituximab versus cladribine using observational data from the Norwegian MS Registry and Biobank at two university hospitals with different treatment strategies. Cumulative incidence and risk differences (RDs) were estimated using weighted Kaplan-Meier estimators, adjusting for baseline covariates. The primary outcome was magnetic resonance imaging (MRI) disease activity, with secondary outcomes including relapses and safety.

**Results:**

The study included 285 patients, 159 receiving rituximab and 126 cladribine, with a median follow-up of 4.5 years (interquartile range (IQR): 4.2–4.7). The 4-year risk of new MRI disease activity was 17% (95% confidence interval (CI): 11–23) for rituximab-treated patients and 57% (95% CI: 44–66) for cladribine-treated patients, yielding an RD of 40 percentage-points (95% CI: 28–50). The 4-year RD for relapse was 12 percentage-points (95% CI: 4–19). The incidence of hospitalizations related to potential adverse events was 6.0 per 100 person-years for rituximab and 4 per 100 person-years for cladribine.

**Conclusion:**

These findings suggest that rituximab has superior effectiveness compared to cladribine during a median follow-up of 4.5 years.

## Introduction

High-efficacy therapies have improved clinical and radiological outcomes for patients with relapsing–remitting multiple sclerosis (RRMS).^1^ Despite their extensive use,^2^ head-to-head randomized controlled trials (RCTs) are lacking, leaving the question of which therapy is most effective unanswered.

Rituximab, an anti-CD20-antibody used off-label, and cladribine, a purine analogue, have been widely used in Norway as first- and second-line treatments for RRMS. Randomized trials have demonstrated their high efficacy in reducing magnetic resonance imaging (MRI) disease activity and improving clinical outcomes.^3–5^ However, the lack of comparative studies has led to variations in treatment strategies across centers, influenced by factors such as physician preferences, hospital policies on off-label use of rituximab, funding availability, risk tolerance, and convenience. This was evident at two university hospitals in Norway during 2018 and 2019 that created a natural experiment with two parallel cohorts: one predominantly treated with rituximab and the other with cladribine. This historical regional difference in MS treatment strategies, combined with a nationwide MS registry capturing data from over 85% of patients with MS, provided a unique opportunity to evaluate real-world long-term treatment effectiveness in two comparable cohorts.

To compare the effectiveness and safety of rituximab and cladribine, we emulated a target trial, a methodological approach to explicitly target the parameters from randomized trials when analyzing observational data that improve transparency and minimize the chance of self-inflicted biases in observational studies.^6^ The primary outcome was MRI disease activity and the secondary outcomes included relapses, disability progression, and safety, with a median follow-up of 4.5 years.

## Methods

### Design

We assessed treatment effectiveness in patients with RRMS using two population-based parallel cohorts, naturally created at two Norwegian university hospitals with different treatment strategies. Haukeland University Hospital (HUH) in Bergen predominantly treated patients with off-label rituximab, while Oslo University Hospital (OUH) in Oslo primarily used on-label cladribine. Since these two hospitals were exclusive healthcare providers for distinct geographical regions, treatment decisions for individual patients were largely determined by their residential address rather than personal factors. We used a target trial emulation framework to design the study, with the key protocol components outlined in Supplemental eTable 1.

### Study population

Patients included were identified from the Norwegian MS Registry and Biobank (NMSRB), with clinical and imaging data collected from routine patient care follow-ups. Data from the patient records were reviewed to ensure complete registration of covariates, exposures, and outcomes in the registry prior to data extraction. Since adverse events were infrequently reported in the NMSRB, hospitalizations were separately registered from patients’ hospital records by experienced neurologists. In addition, serum samples from a nationwide COVID-19 vaccine response study were included and assessed for biomarker analyses.^7,8^

Patients were eligible if they were ⩾18 years old, had a diagnosis of RRMS, and had received at least one treatment with either cladribine at OUH or rituximab at HUH between 15 May 2018 and 15 October 2019. Patients were excluded if they had a progressive MS disease, were previously treated with either drug, or lacked reported data on baseline or consecutive MRI examinations. An individual’s baseline was defined as the date of initiation of treatment with cladribine or rituximab. The most recent MRI conducted within 12 months prior to baseline, or up to 1 month following, was considered the baseline MRI. The data cut-off date, also representing end of follow-up, was 31 August 2023. Harmonization efforts in Norway in 2015 and 2016 led to standardized MRI protocols for MS evaluation at both centers, including three-dimensional (3D) or sagittal and coronal FLAIR, T1, and T2 sequences in accordance with current national recommendations.^9^ Brain MRI was performed at baseline and typically repeated after 3–6 months after treatment initiation to establish a re-baseline, followed by routine annual imaging. Spinal cord MRI was included as part of the diagnostic work-up or conducted when clinically indicated.

### Exposures

All patients were treated (exposed) with either rituximab or cladribine at baseline, hereafter referred to as index therapy. The standard rituximab dosing at HUH was 1000 mg iv. at initiation, followed by 500 mg every 6 months.^10^ The standard regimen of cladribine tablets was a cumulative dose of 3.5 mg/kg over the first 2 years with cycles of 4 to 5 days twice each year.^4^

### Outcomes

Primary outcome was the time to new MRI disease activity, defined as new T2-lesions on brain or spinal cord MRI compared to baseline MRI. Secondary outcomes were time to the first relapse, defined as an acute or subacute MS episode lasting ⩾24 hours with or without recovery and unrelated to infection or fever;^11^ time to discontinuation of treatment or a third dose of cladribine; confirmed disability progression (CDP), defined as an increase of at least 1.5 points from a baseline of 0, 1.0, or more points from a baseline of 1.0 to 5.5, and at least 0.5 points from a baseline of ⩾6, confirmed by a sustained increase of the same or higher score at the 6 months follow-up visit or later; confirmed disability improvement (CDI), defined as any decrease of Expanded Disability Status Scale (EDSS) score confirmed by a sustained decrease of the same or lower score at the 6 months follow-up visit or later; and the proportion of patients with no evidence of disease activity (NEDA-3), defined as no MRI disease activity, no relapse, and no worsening in EDSS score. Additional secondary outcomes were serum levels of neurofilament light chain (NfL) and glial fibrillary acidic protein (GFAP) in the last available sample from each patient, measured with the Quanterix Simoa Neurology 2-Plex B Assay Kit; and hospitalizations related to possible adverse events (infections, malignancies, or cardiac arrythmias) and deaths during index therapy. Multiple hospitalizations associated with the same type of adverse event in one patient were counted multiple times, except for malignancies, which were orderly counted as one event.

### Covariates

Baseline covariates included in the adjusted analyses were age, sex, disease duration, number of previous disease-modifying therapies (DMTs), total number of T2-lesions, EDSS score, relapses within 12 months prior to baseline, MRI lesion activity within 12 months prior to baseline, time between the most recent MRI conducted and baseline, and reasons for discontinuing the last DMT prior to baseline (Table 1).

**Table 1.** Baseline Characteristics Before and After Weighting.

Characteristic	Unweighted numbers		Weighted proportions	
	Rituximab *N* = 159	Cladribine *N* = 126	Rituximab	Cladribine
Age, mean (SD), y	42 (11)	41 (11)	42.1 (11.2)	42.6 (11.3)
Sex				
Female	118 (74%)	95 (75%)	75.7%	73.1%
Male	41 (26%)	31 (25%)	24.3%	26.9%
Disease duration, y				
Mean (SD) Median (IQR)	9 (9) 6 (13)	10 (9) 8 (11)	9.2 (9.4) 6.1 (13.7)	9.2 (9.0) 7.7 (11.7)
No. of previous DMTs				
0	73 (46%)	35 (28%)	38.5%	37.2%
1	31 (19%)	42 (33%)	24.1%	24.5%
2	31 (19%)	25 (20%)	20.0%	20.9%
3	14 (9%)	14 (11%)	10.8%	10.0%
4–6	10 (6%)	10 (8%)	6.8%	7.4%
MRI, T2 lesion load, n				
0–5	20 (13%)	21 (17%)	13.2%	13.8%
6–10	46 (29%)	14 (11%)	22.3%	22.2%
>10	93 (58%)	91 (72%)	64.5%	64.0%
Unknown	0 (0%)	0 (0%)	0%	0%
Disability, EDSS score^a^				
Mild, 0–2	41 (26%)	30 (24%)	24.3%	22.3%
Moderate, 2.5–5	14 (9%)	16 (13%)	10.0%	9.9%
Severe, 5.5 and higher	4 (2.5%)	1 (1%)	2.1%	4.6%
Unknown	100 (63%)	79 (63%)	63.7%	63.2%
Relapse within 12 months before baseline	87 (55%)	46 (37%)	47.5%	49.0%
MRI disease activity within 12 months before baseline	110 (69%)	96 (76%)	70.2%	69.0%
Reason for discontinuing the last DMT before baseline				
No DMTs prior to index treatment	73 (46%)	35 (28%)	38.5%	37.2%
Treatment failure	37 (23%)	30 (24%)	23.8%	21.3%
Side effects	29 (18%)	28 (22%)	21.1%	20.7%
Other reasons^b^	20 (13%)	32 (25%)	16.5%	20.5%
Unknown	0 (0%)	1 (1%)	0%	0.3%
Time between the most recent MRI and baseline, median (IQR), d	31 (58)	57 (63)	33 (67)	52 (68)

Abbreviations: y, years; no, number; d, days; DMT, disease-modifying therapy; MRI, magnetic resonance imaging; mo, months; EDSS, Expanded Disability Status Scale.

a The last reported non-relapse EDSS score within the year prior baseline.

b Patient’s decision, family planning, and other reasons.

### Statistical analysis

Cumulative incidence of the outcomes was estimated using Kaplan–Meier estimators. Adjustments for baseline covariates were performed using stabilized inverse probability of treatment (propensity score) weights, estimated using logistic regression.^12^ Details on statistical, subgroup, and sensitivity analysis are provided in eMethods.

## Results

### Study population and follow-up

**Figure 1.**
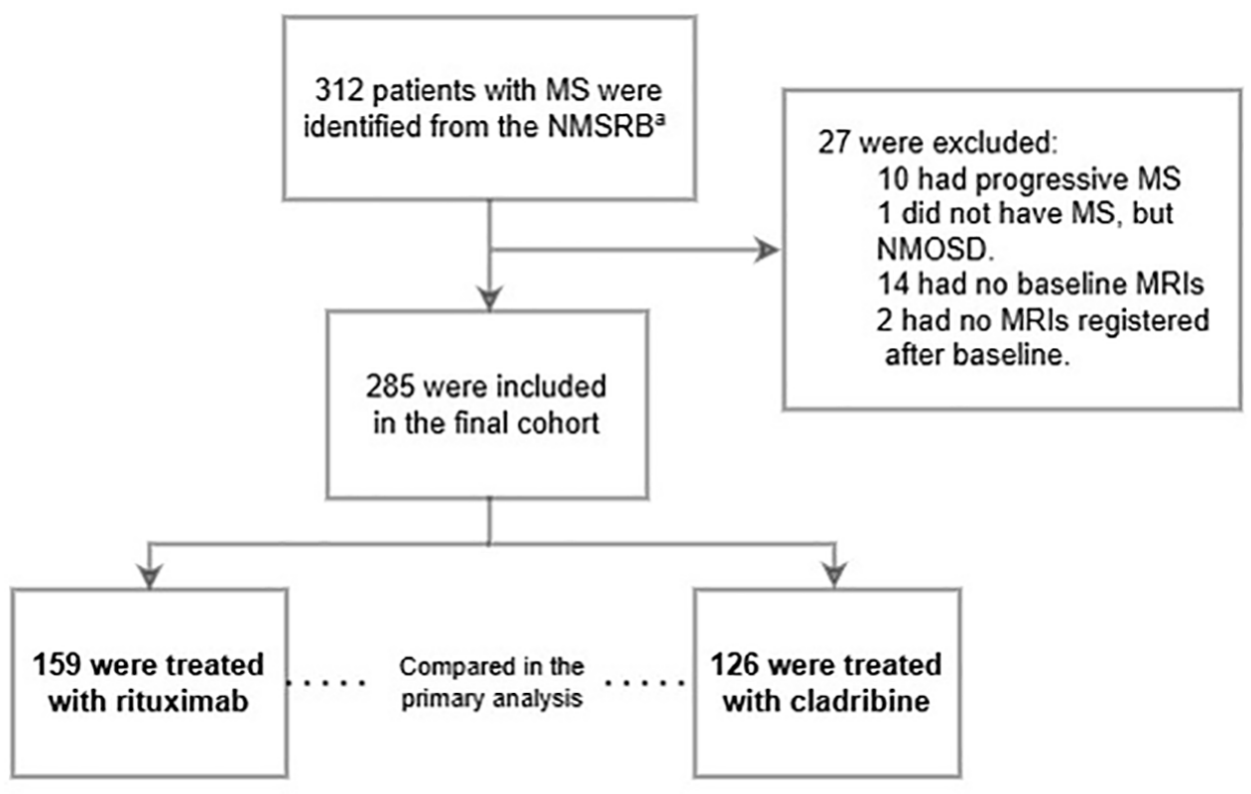
Flowchart of study inclusion. ^a^Patients who initiated treatment with rituximab at Haukeland University Hospital (HUH) or cladribine at Oslo University Hospital (OUH) between 15 May 2018 and 15 October 2019. Abbreviations: MS, multiple sclerosis; NMSRB, Norwegian MS Registry and Biobank; NMOSD, neuromyelitis optica spectrum disorder; MRI, magnetic resonance imaging; OUH, Oslo University Hospital; HUH, Haukeland University Hospital; EDSS, Expanded Disability Status Scale.

We included 285 patients with RRMS; 159 patients were treated with rituximab and 126 were treated with cladribine (Figure 1). Baseline characteristics are presented in Table 1. After inverse probability weighting, covariates were balanced between the treatment groups, except in patients with severe disability and those with “other reasons” for discontinuing the previous DMT (Supplemental eFigure 1). The median follow-up period was 4.5 years (interquartile range, IQR: 4.2–4.7). Between baseline and end of study observation period, a median of five MRIs was conducted in both treatment groups (IQR of 4–5 for rituximab-treated and 5–6 for cladribine-treated).

### Primary outcome

**Figure 2.**
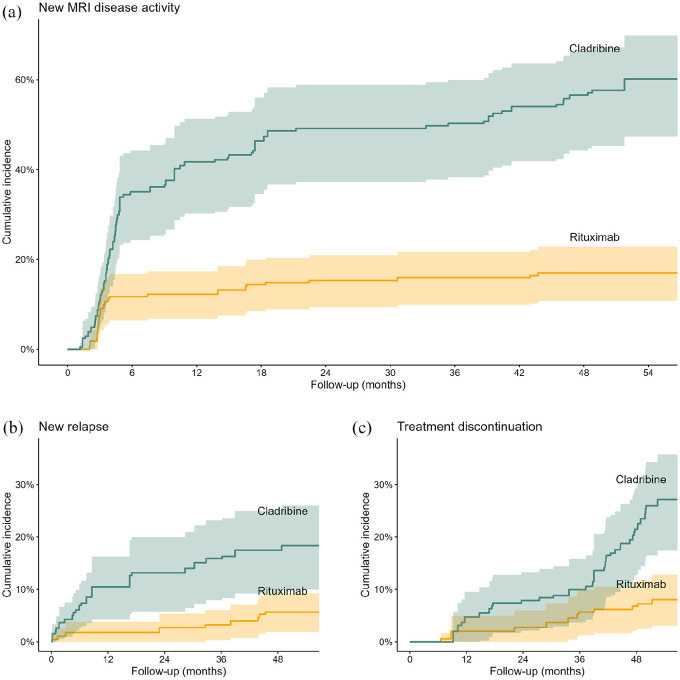
Cumulative incidence of outcomes. Cumulative incidence of outcomes up to 5 years after index treatment initiation. Shaded areas represent 95% confidence intervals. (a) Time to new MRI disease activity, defined as new T2-lesions on brain or spinal cord MRI, compared to baseline MRI. (b) Time to first relapse. (c) Time to treatment discontinuation or a third dose of cladribine, among cladribine-treated patients. Adjustments for baseline covariates (age; sex; disease duration; number of previous DMTs; number of T2-lesions on MRI; EDSS score; relapses within 12 months prior to baseline; MRI lesion activity within 12 months prior to baseline; time from baseline MRI to baseline; and reasons for discontinuing the last DMT prior to the index therapy) were performed using stabilized inverse probability of treatment (propensity score) weights, estimated using logistic regression. Abbreviations: MRI, magnetic resonance imaging; DMTs, disease-modifying therapies; EDSS, Expanded Disability Status Scale.

The risk of new MRI disease activity was lower for patients treated with rituximab compared to patients treated with cladribine (weighted log-rank test, *p* < 0.0001, Figure 2). The 4-year risk of new MRI disease activity was 17% (95% confidence interval (CI): 11–23) for rituximab-treated patients and 57% (95% CI: 44–66) for cladribine-treated patients. The risk difference (RD) at 6 months, 2 years, and 4 years after treatment initiation was 23 percentage-points (95% CI: 15–35), 34 percentage-points (95% CI: 23–43) and 40 percentage-points (95% CI: 28–50). The risk ratio (RR) at 6 months, 2 years, and 4 years after treatment initiation was 0.34 (95% CI: 0.19–0.51), 0.31 (95% CI: 0.21–0.47), and 0.30 (95% CI: 0.20–0.44) for patients treated with rituximab compared to cladribine (Supplemental eTables 2 and 3, and Table 2). During the 4.5 years of follow-up, the patients treated with rituximab were free of new MRI disease activity for a mean of 16.8 months (95% CI: 10.7–23.4) longer than those treated with cladribine.

**Table 2.** Comparative Effectiveness 4 Years After Initiation of Rituximab (*n* = 159) and Cladribine (*n* = 126) in Patients With Multiple Sclerosis.

Outcome	Number of events		4-year risk (95% CI)		Risk difference^a^ (95% CI)	Risk ratio^a^ (95% CI)
	Rituximab	Cladribine	Rituximab	Cladribine	*Percentage-points*	
New MRI activity^b^	28	72	17% (11–23)	57% (44–66)	39.6 (28.4–49.8)	0.30 (0.20–0.44)
New relapse	9	21	5.7% (1.9–9.3)	17% (9.2–25)	11.8 (4.0–18.9)	0.32 (0.12–0.68)
Treatment discontinuation^c^	9	28	6.8% (2.1–11)	21% (13–29)	14.7 (8.8–25.4)	0.32 (0.11–0.51)

Abbreviations: CI, confidence interval; MRI, magnetic resonance imaging.

a Adjusted for age; sex; disease duration; number of previous disease-modifying therapies (DMTs); number of T2-lesions on MRI; EDSS score; relapses within 12 months prior to baseline; MRI lesion activity within 12 months prior to baseline; time from baseline MRI to baseline; and reasons for discontinuing the last DMT prior to the index therapy.

b New MRI disease activity, defined as new T2-lesions on brain or spinal cord MRI compared to baseline MRI.

c Treatment discontinuation or third dose of cladribine, among cladribine-treated patients.

### Secondary outcomes

The risk of relapses 4 years after treatment initiation was lower for patients treated with rituximab (6% [95% CI: 2–9]) than for patients treated with cladribine (17% [95% CI: 9–25], *p* = 0.0012), with an RD of 12 percentage-points (95% CI: 4–19) and an RR of 0.32 (95% CI: 0.12–0.68; Table 2). After 4 years, fewer rituximab-treated patients had discontinued their therapy (7% [95% CI: 2–11]) compared to those treated with cladribine (21% [95% CI: 13–29], *p* < 0.0001), with an RD of 15 percentage-points (95% CI: 9–25) and an RR of 0.32 (95% CI: 0.11–0.51). The main reasons for discontinuing index therapy or receiving a third dose of cladribine were side effects for rituximab and disease activity for cladribine (Supplemental eTable 4). Nine cladribine-treated patients received a third dose, while 26 switched to another DMT.

EDSS scores were available at baseline and during follow-up for 96 patients (34%). After 4 years, there was no significant difference in the cumulative incidence of CDP between treatment groups (rituximab, 9% [95% CI: 2–16]; cladribine, 19% [95% CI: 3–32]; *p* = 0.32), but a higher proportion of rituximab-treated patients had CDI (21% [95% CI: 9–32]) compared to those receiving cladribine (4% [95% CI: 0–9]; *p* = 0.026), corresponding to an RD of 18 percentage-points (95% CI: 4–29) and an RR of 0.16 (95% CI: 0.0–0.7; Supplemental eFigure 2). The proportion with NEDA-3 status was higher in rituximab-treated patients compared to cladribine-treated patients (adjusted odds ratio (OR) = 15.9, 95% CI: 3.8–92.4, *p* = 0.0006; Supplemental eFigure 2).

NfL and GFAP were available for analysis in 133 patients (47%) using serum samples collected 1.7 to 4.2 years after baseline. NfL levels did not differ statistical significantly between the treatment groups (5.6 pg/mL in the rituximab-treated vs 6.9 pg/mL in the cladribine-treated, *p* = 0.18). GFAP levels were lower in the rituximab-treated patients (62.6 pg/mL) compared to the cladribine-treated patients (87.8 pg/mL, *p* = 0.021; Supplemental eFigure 3).

Information about adverse events was available for 284 of 285 patients (99.6%). The incidence of hospitalizations related to possible adverse events during rituximab therapy was 6.0 per 100 person-years versus 4.1 per 100 person-years during cladribine therapy. The most frequent reason for hospitalization was COVID-19 (including post-acute COVID-19 syndrome) in the rituximab-treated patients with the highest incidence in 2021 and 2022, while in the cladribine-treated patients, it was other respiratory diseases (Table 3 and Supplemental eTable 5). No patients died during the follow-up period.

**Table 3.** Hospitalizations Related to Possible Adverse Events.

	Therapy	
	Rituximab *N* = 158 697 PY	Cladribine *N* = 126 516 PY
Hospitalizations due to any possible adverse drug event, n	42	21
Patients with ⩾1 hospitalization due to any possible adverse drug event, n	26	12
Hospitalizations per 100 person-years	6.0	4.1
Adverse events per ICD-10 categories, *n*		
Infectious and parasitic diseases	3	3
COVID-19 and post-acute COVID-19 syndrome	16	2
Diseases of the circulatory system	4	2
Diseases of the digestive system	0	2
Diseases of the genitourinary system	3	4
Diseases of the musculoskeletal system and connective tissue	0	1
Diseases of the nervous system	0	1
Diseases of the respiratory system	13	5
Injury, poisoning, and certain other consequences of external causes	1	0
Neoplasms	1	1
Symptoms, signs, and abnormal clinical and laboratory findings, not elsewhere classified	1	0

Multiple hospitalizations for the same type of adverse event in one patient were counted multiple times, except for malignancies, which were orderly counted as one event.

Abbreviations: PY, person-years; ICD, International Classification of Diseases.

### Subgroup and sensitivity analysis

The cumulative incidence curves were similar across subgroups defined by age, sex, and treatment history (Supplemental eFigure 4) and remained consistent for patients switching from therapies known to give a high risk of rebound activity (fingolimod and natalizumab) and from the other DMTs (data not shown). The re-baseline MRIs were conducted a median of 3.1 months (IQR: 2.8–3.6) after the initiation of rituximab and 3.6 months (IQR: 2.9–4.6) after the initiation of cladribine. Analyses using data from re-baseline MRI as the initial MRI (instead of baseline MRI) showed that patients who were treated with rituximab had a lower risk of new MRI disease activity, compared to those who were treated with cladribine (*p* < 0.0001). The 4-year risk after re-baseline was 7% (95% CI: 3–11) for the rituximab-treated patients and 37% (95% CI: 26–47) for the cladribine-treated patients, yielding an RD of 30 percentage-points (95% CI: 22–41, Supplemental eFigure 5). Results from the sensitivity analysis using a baseline set 18 months after treatment initiation confirmed that the difference in lesion incidence extends beyond the first 18 months (Supplemental eFigure 6).

When comparing new MRI disease activity between the two hospitals with different treatment strategies, including all patients regardless of type of initial DMT, we included 436 patients: 193 at HUH and 243 patients at OUH. At HUH, 80% of the patients started on rituximab and 1% started on cladribine, while at OUH, 49% started cladribine and 11% started rituximab (Supplemental eTable 7). Patients treated at HUH had lower risk of new MRI disease activity, compared to patients treated at OUH (*p* < 0.0001, Supplemental eFigure 7). When comparing all patients who received rituximab with all patients who received cladribine, independent of hospital, rituximab-treated patients had lower risk of new MRI disease activity (Supplemental eFigure 8).

## Discussion

In this observational comparative study with a median follow-up of 4.5 years, rituximab showed superior effectiveness compared to cladribine in reducing new MRI disease activity, our primary outcome. We also observed fewer relapses, less treatment discontinuation, and improved disability outcomes with rituximab. Hospitalizations related to adverse events were comparable between the treatment groups.

Our results on new MRI disease activity, a sensitive and objective marker of disease activity routinely used in clinical practice,^11,13^ were consistent with those reported from other studies. In the RIFUND-MS trial, which randomized treatment-naïve patients to rituximab or dimethyl fumarate, 21% of the rituximab-treated patients had new lesions on MRI after two years,^3^ the same proportion as for our subgroup of treatment-naïve patients who received rituximab. In the placebo-controlled CLARITY and the CLARITY Extension trial, 65.6% of the patients who received two courses of cladribine had active T2-lesions in the following 2 years.^14,15^ In our cohort, 57% (95% CI: 44–66) had new lesions 4 years after cladribine initiation. An ongoing randomized trial (NOR-MS, NCT04121403) is comparing rituximab and cladribine over 2 years with new MRI T2-lesions as the primary outcome, but results are not yet available.

The marked difference in risk of MRI disease activity that emerged within the first 6 months between rituximab and cladribine suggests a delayed treatment effect of cladribine. Rapid treatment effect is particularly crucial when there is a high risk of continued or rebound disease activity, as described after discontinuation of fingolimod and natalizumab.^16,17^ We have previously reported a higher risk of rebound among patients who switched from fingolimod to cladribine (21%, 7/33), compared to those who switched to rituximab (0/40).^18^ However, the difference in effectiveness in our study was not limited to the first months after treatment initiation or to prior DMT, as the difference persisted in analyses starting at re-baseline MRI or 18 months after treatment initiation, and in the subgroup of treatment-naïve patients.

The 4-year risk of relapse was lower for rituximab compared to cladribine in our study. This is consistent with a recent registry-based publication from the MSBase, which found a lower relapse rate among patients treated with ocrelizumab compared to those treated with cladribine (0.05 vs 0.09, *p* = 0.008).^19^ However, we observed a lower cumulative incidence of relapses for both rituximab and cladribine, compared to the previous RCTs.^3–5^ This finding may be due to differences in patient composition, since the original rituximab and cladribine studies were performed a decade before the present study,^4,5^ or to a more stringent use of the relapse definitions and differences in reporting of relapses, as also suggested in a report from the Swedish MS registry.^20^

The 4-year risk of CDP did not differ between treatment groups; however, patients treated with rituximab had a higher chance of CDI, aligned with previous findings.^19^ Serum NfL, a biomarker of axonal damage and acute inflammation,^21^ did not differ significantly between the treatment groups in our study. On the other hand, serum GFAP, an intermediate filament in astrocytes and proposed biomarker for disease progression independent of relapse activity,^21,22^ was found at lower levels in patients who were treated with rituximab compared to those who were treated with cladribine. While this finding correlates with the observed effect on disability, it should be interpreted cautiously, as no longitudinal samples were available, and the role of serum GFAP in MS is not yet fully established.^22^

The observation period of our study included the years of the COVID-19 pandemic. During this time, infections were particularly concerning for patients treated with anti-CD20 antibodies, as they had an attenuated humoral vaccine response.^7^ Although studies have reported a high relative risk of hospitalization and severe COVID-19 infection associated with anti-CD20 treatment compared to other DMTs,^23–25^ the absolute risk of severe COVID-19 infections in Norway has been low.^26^ In our study, COVID-19 was the most frequent adverse event related to hospitalizations among the patients treated with rituximab, a striking difference to those on cladribine (16 vs 2). While this reflects a more profound immunomodulatory effect of rituximab, it can also be related to an increased awareness and early healthcare response for rituximab-treated patients, as they were eligible for early antiviral treatment upon COVID-19 disease. The number of hospitalizations potentially related to adverse events other than COVID-19 was similar for patients treated with rituximab and cladribine in our study. This aligns with other reports that have indicated the same risk of infections for rituximab as other high-efficacy therapies and in the RCTs, where risks of serious infections were similar between the anti-CD20 treatment arms and the comparators (placebo, interferon-beta 1a, teriflunomide, and dimethyl fumarate).^3,5,27–30^ However, a Swedish observational study reported a higher risk of serious infections in patients with MS treated with rituximab compared to those treated with natalizumab, fingolimod, interferon beta, and glatiramer acetate.^31^ Future studies may shed light on the relative risk of infections with different DMTs, and especially for long-term use of anti-CD20 therapies.

### Strengths and limitations

Our study population emerged from a near-randomly occurring difference in treatment preferences between two hospitals, where treatment was primarily determined by patients’ residential addresses. While it is impossible to distinguishing treatment allocation from local assessment practices, Norway’s homogeneous population, standardized healthcare system, and national clinical guidelines for MS management likely minimize this issue. In addition, the high-coverage Norwegian MS Registry provided a unique opportunity to assess real-world long-term effectiveness, including a broader age range and without excluding patients with comorbidities, knowledge that is usually not captured in RCTs. To further minimize common biases in observational studies, we applied the target trial framework, which allowed us to structure the study in a way that facilitates causal inference.

The main limitation of this study is the lack of randomization. We cannot account for all potential confounders, such as smoking status, body mass index, comorbidity, ethnic background, or other unmeasured confounders. Nonetheless, the thorough sensitivity and sub-analyses, and similarity of our estimates to those reported from previous RCTs, suggest that our findings are reliable. The scarcity of EDSS data represents another limitation, and these results should be interpreted cautiously. However, they align with the MRI and relapse activity findings. Another limitation is that serum NfL and GFAP were only measured at one time point per patient, with samples collected randomly throughout the treatment course, preventing evaluation on changes from treatment start and during therapy. Finally, the time between the baseline MRI scan and the initiation of index therapy varied among the patients, and therefore, new lesions reported at the re-baseline MRI could have appeared before therapy initiation. However, the sensitivity analyses of the primary outcome, analyzing new MRI disease activity after the re-baseline MRI examination and after 18 months, confirmed the consistent pattern of difference in treatment effectiveness between the groups.

In summary, our propensity score-weighted comparative study with a median follow-up of 4.5 years shows that rituximab has superior effectiveness over cladribine in controlling MRI disease activity. Rituximab was also associated with fewer relapses and disability improvement, while safety outcomes were comparable. These high-quality long-term data, especially on MRI and safety outcomes, provide valuable insights for treatment decisions. However, RCTs are warranted to confirm these findings in fully comparable cohorts and to capture more comprehensive disability outcomes.

## Supplemental Material

sj-docx-1-msj-10.1177_13524585251342727 – Supplemental material for Comparative effectiveness of rituximab and cladribine in relapsing–remitting multiple sclerosis: A target trial emulation Supplemental material, sj-docx-1-msj-10.1177_13524585251342727 for Comparative effectiveness of rituximab and cladribine in relapsing–remitting multiple sclerosis: A target trial emulation by Brit Ellen Rød, Einar A Høgestøl, Øivind Torkildsen, Kjetil Bjørnevik, Jon Michael Gran, Mathias H Øverås, Marton König, Kjell-Morten Myhr, Stig Wergeland and Gro O Nygaard in Multiple Sclerosis Journal
