# Supplementary material for: Comparative effectiveness of rituximab and cladribine in relapsing–remitting multiple sclerosis: A target trial emulation
Source: Mult Scler. 2025 May 26;31(8):975–84. doi: 10.1177/13524585251342727 (PMC12228892; doi:10.1177/13524585251342727)
Supplement: sj-docx-1-msj-10.1177_13524585251342727 – Supplemental material for Comparative effectiveness of rituximab and cladribine in relapsing–remitting multiple sclerosis: A target trial emulation [file sj-docx-1-msj-10.1177_13524585251342727.docx]

**Supplementary Appendix**

Supplementary data to BE. Rød, EA. Høgestøl, Ø. Torkildsen, K. Bjørnevik, JM. Gran, MH. Øverås, M. König, K-M Myhr, S. Wergeland, GO. Nygaard **Comparative Effectiveness of Rituximab and Cladribine in Relapsing-Remitting Multiple Sclerosis: A Target Trial Emulation** submission to *Multiple Sclerosis Journal.*

**eMethods: STATISTICAL, SUBGROUP AND SENSITIVITY ANALYSES**

**FIGURES**

**eFigure 1.** Absolute standardized differences before and after applying inverse probability weighting.

**eFigure 2.** Cumulative incidence of confirmed disability progression and improvement, and NEDA-3 status at end of follow-up**.**

**eFigure 3.** sNfL and sGFAP.

**eFigure 4.** Cumulative incidence new MRI disease activity in subgroups.

**eFigure 5.** Cumulative incidence of new MRI disease activity from re-baseline MRI.

**eFigure 6.** Cumulative incidence of new MRI disease activity with baseline set to 18 months after treatment initiation.

**eFigure 7.** Cumulative incidence of new MRI disease activity by treatment strategy cohorts.

**eFigure 8.** Cumulative incidence of new MRI disease activity by therapy, independent of hospital

**TABLES**

**eTable 1.** Specification and emulation of the target trial.

**eTable 2.** Estimated comparative effectiveness after 6 months.

**eTable 3.** Estimated comparative effectiveness after 24 months.

**eTable 4.** Reasons for discontinuation or third dose of cladribine.

**eTable 5**. Timing of adverse events after treatment initiation.

**eTable 6.** Baseline variables by treatment strategy cohort.

**Statistical Analysis**

The occurrence of outcome events was described using plots of cumulative incidence (risk). In the absence of competing events, as no patients died, cumulative incidence was estimated using the Kaplan-Meier estimator. Absolute and relative risks differences between groups were estimated at 6 months, 2 years, and 4 years. Adjustments for baseline covariates were performed using stabilized inverse probability of treatment (propensity score) weights, estimated using logistic regression.^1^ Given the assumptions that the included baseline covariates sufficiently adjust for confounding, and that there is positivity (overlap) between the two treatment groups, group comparisons using the weighted data will correspond to the average treatment effect (ATE) of initiating rituximab versus initiating cladribine in the eligible patient population. Such effects of initiating treatments is sometimes referred to as an observational analogue of an intention-to-treat effect.^1^ The treatment groups were compared on adjusted mean time free of new MR disease activity over 4.5 years (the weighted restricted mean survival time, RMST, and weighted log-rank test for difference).^2^ Percentile-based 95% confidence intervals (CI) for all estimates were calculated using non-parametric bootstrap with 1000 bootstrap samples. Baseline covariate balance was assessed using absolute standardized mean differences between the groups, with a difference of up to 0.1 considered acceptable.

Proportions of no evidence of disease activity (NEDA-3, limited to patients with available EDSS scores) were compared using logistic regression. Neurofilament light chain (NfL) and glial fibrillary acidic protein (GFAP) levels were log-transformed and compared between the treatment groups using linear regression. Geometric means were calculated by back-transforming log-transformed values. All analyses were adjusted for baseline covariates (described in Table 1), and the confidence intervals were calculated at the 2-sided 95% level. The statistical analyses were conducted using R, version 4.1.2 (R Foundation for Statistical Computing), with the packages cobalt, WeightIt and survival. Figures were created in R using ggplot2.

**Subgroup and sensitivity analyses**

For the primary outcome, time to new MRI disease activity, we conducted prespecified subgroup analyses according to the treatment history (treatment-naïve or not), age (<40 or ≥40 years) and sex. As a prespecified sensitivity analysis, we evaluated the primary outcome in all patients at both hospitals who initiated any disease modifying therapies (DMT) within the specified baseline time interval, defining the first DMT started during the baseline interval as the index therapy. We then compared rituximab versus cladribine irrespective of hospital site. In addition, we included two separate analyses using re-baseline MRI (the first MRI conducted after baseline, typically 3-6 months post-initiation) as baseline and a baseline set at 18 months following treatment initiation.

**REFERENCES**

1. Hernán M and Robins J. Causal Inference: What If. 2020.

2. Stensrud MJ, Aalen JM, Aalen OO and Valberg M. Limitations of hazard ratios in clinical trials. *Eur Heart J* 2019; 40: 1378-1383. DOI: 10.1093/eurheartj/ehy770.

eFigure 1. Absolute standardized mean differences before and after applying inverse probability weighting.


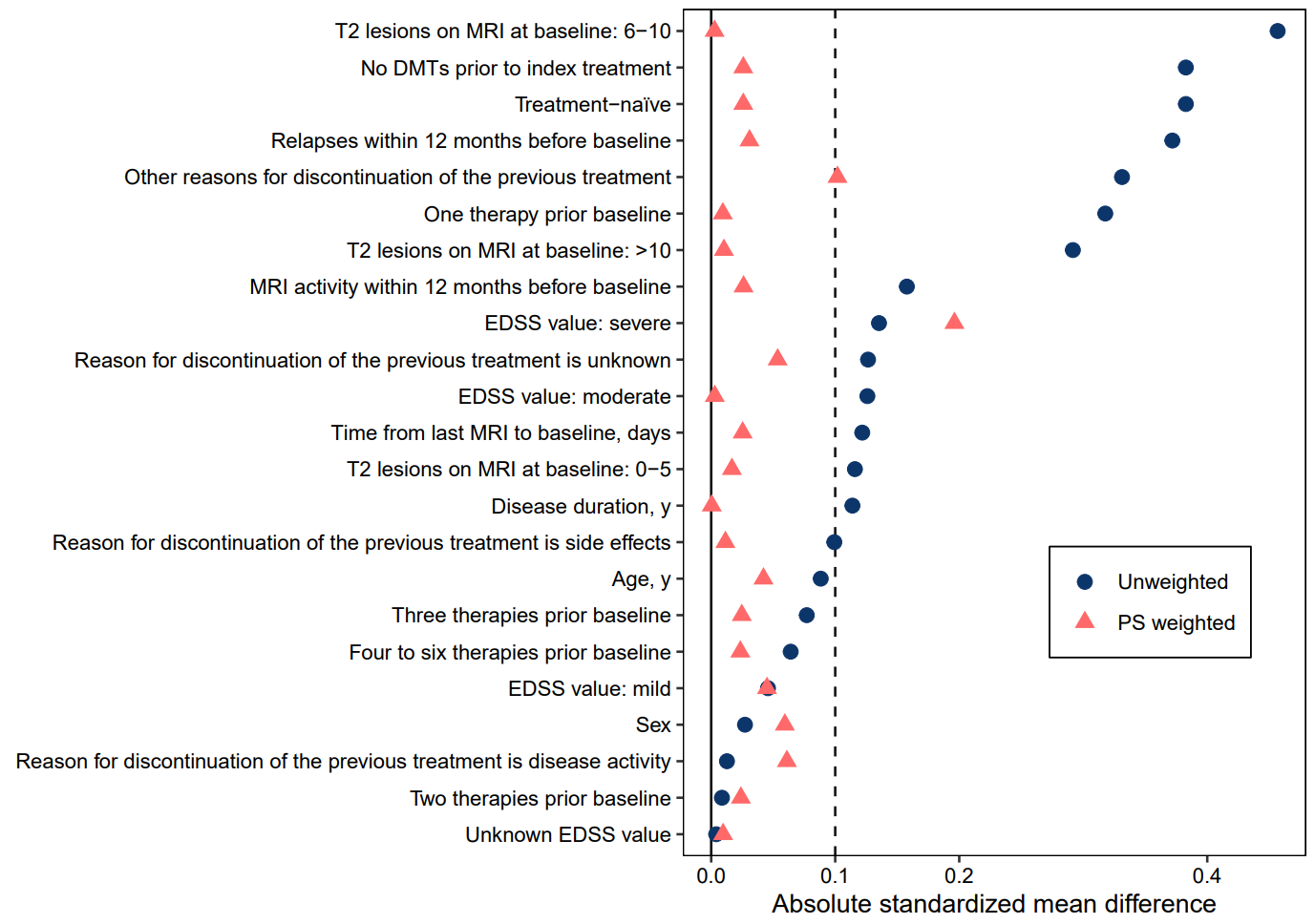


**Abbreviations**: DMTs, disease-modifying therapies; MRI, magnetic resonance imaging; EDSS, the Expanded Disability Status Scale.

eFigure 2. Cumulative incidence of confirmed disability progression and improvement, and NEDA-3 status at end of follow-up.


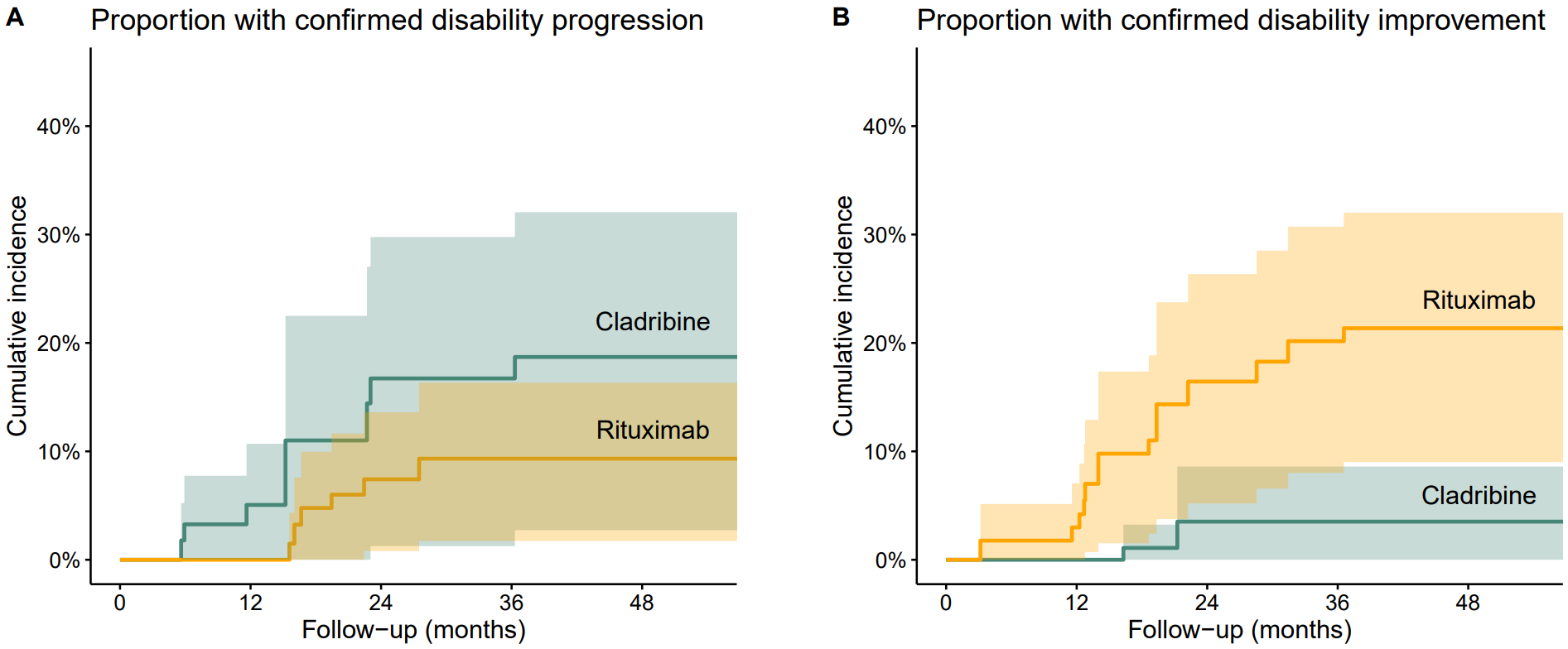


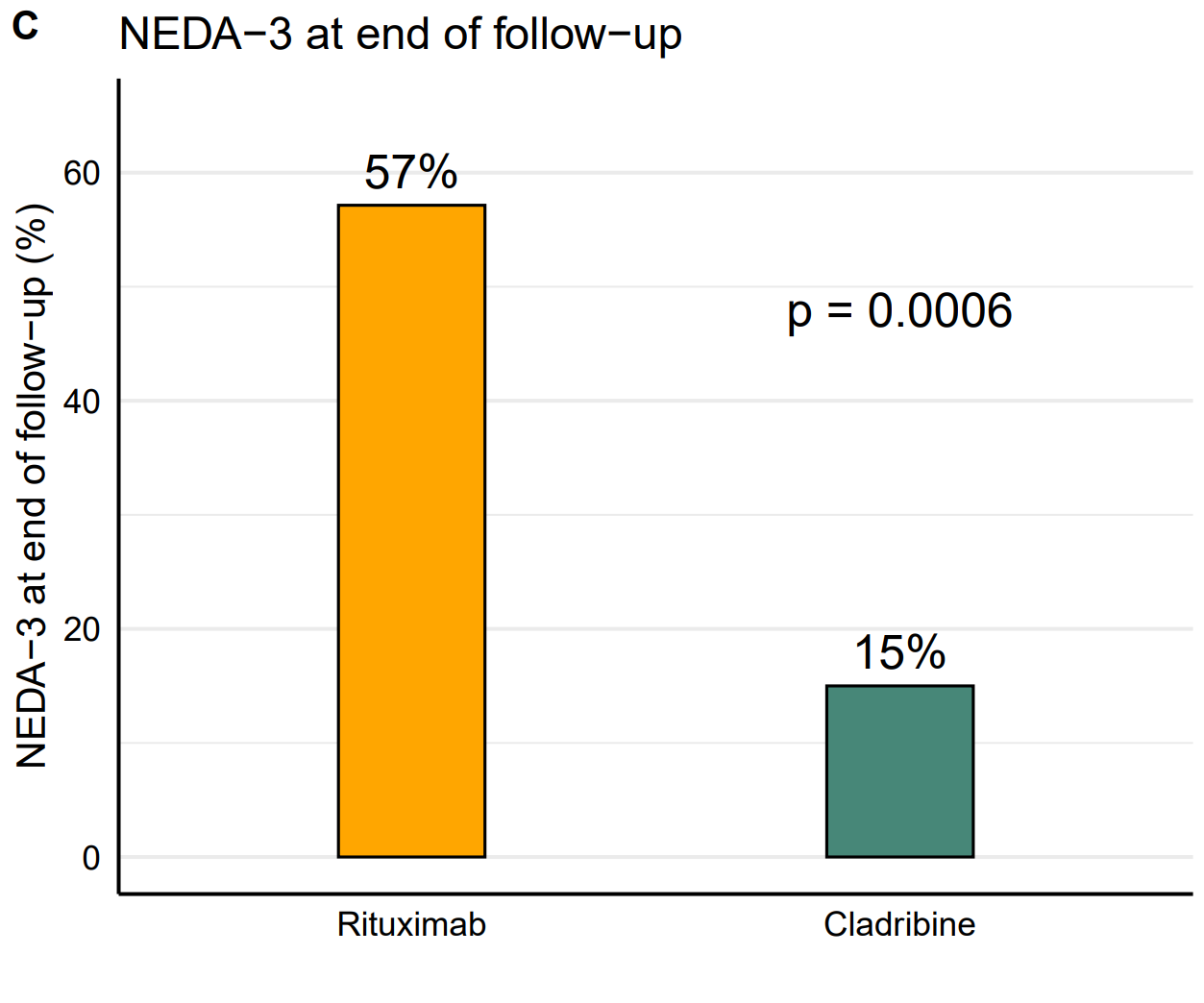


1. Cumulative incidence of confirmed disability progression (CDP) among 96 patients with MS (cladribine, n = 40, rituximab, n = 56) followed for up to 5 years after index treatment initiation. CDP was defined as an increase of at least 1.5 points from a baseline of 0, 1.0 or more points from a baseline of 1.0 to 5.5, and at least 0.5 points from a baseline of ≥6. Progression had to be confirmed by a sustained increase of the same or higher score at the 6-month follow-up visit or later (with a minimum interval of 5 months).
2. Cumulative incidence of confirmed disability improvement (CDI) in the same cohort (cladribine, n = 40, rituximab, n = 56) up to 5 years after index treatment initiation. CDI was defined as any decrease of EDSS score, confirmed by a sustained decrease of the same or lower score at a follow-up visit at the 6-month follow-up visit or later (with a minimum interval of 5 months). Adjustments for baseline covariates (age; sex; disease duration; number of previous DMTs; number of T2-lesions on MRI; EDSS score; relapses within 12 months prior to baseline; MRI lesion activity within 12 months prior to baseline; time from baseline MRI to baseline; and reasons for discontinuing the last DMT prior to the index therapy) were performed using stabilized inverse probability of treatment (propensity score) weights, estimated using logistic regression.
3. No evidence of disease activity (NEDA)-3 status at end of follow-up among 96 patients with MS (cladribine, n = 40, rituximab, n = 56). NEDA-3 was considered patients with no worsening in EDSS score, no new MRI disease activity and no relapses. We used linear regression adjusting for baseline covariates to compare the proportions of patients with NEDA-3 status between the treatment groups. The baseline covariates included in the analyses were age; sex; disease duration; number of previous DMTs; number of T2-lesions on MRI; EDSS score; relapses within 12 months prior to baseline; MRI lesion activity within 12 months prior to baseline; time from baseline MRI to baseline; and reasons for discontinuing the last DMT prior to the index therapy.

**Abbreviations**: EDSS, the Expanded Disability Status Scale; CDP, confirmed disability progression; CDI, confirmed disability improvement; NEDA-3, no evidence of disease activity. DMTs, disease-modifying therapies; MRI, magnetic resonance imaging.

eFigure 3. sNfL and sGFAP


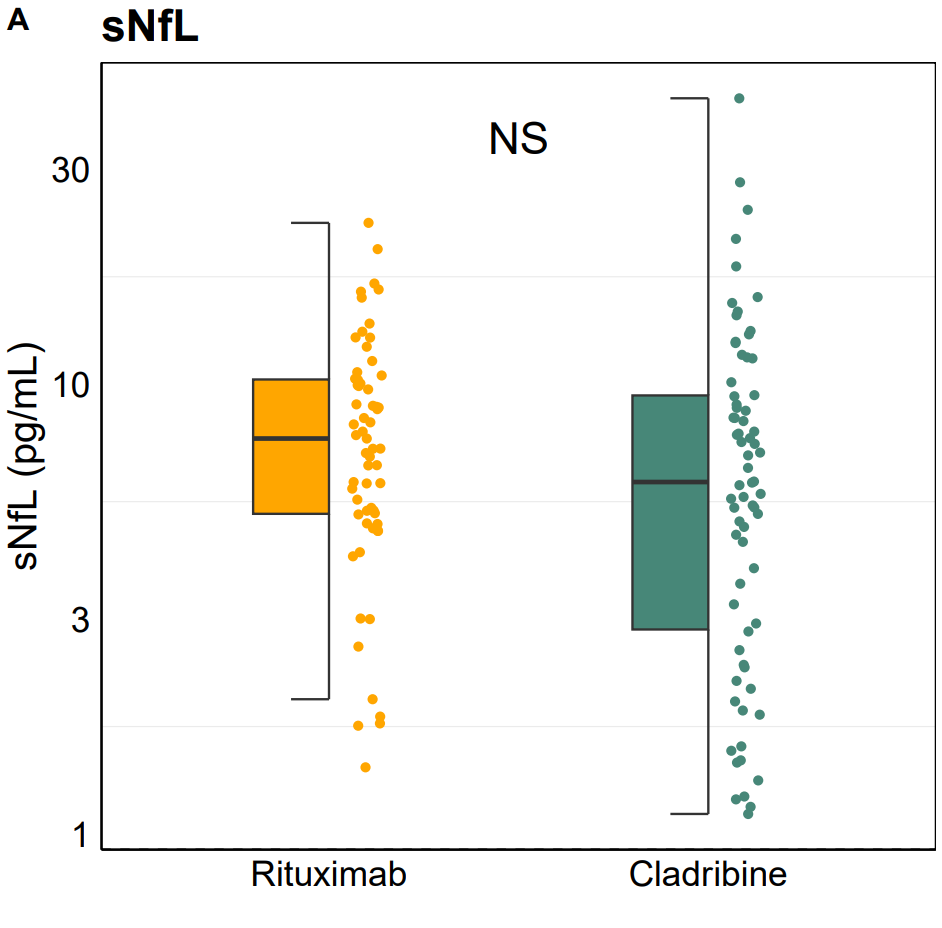

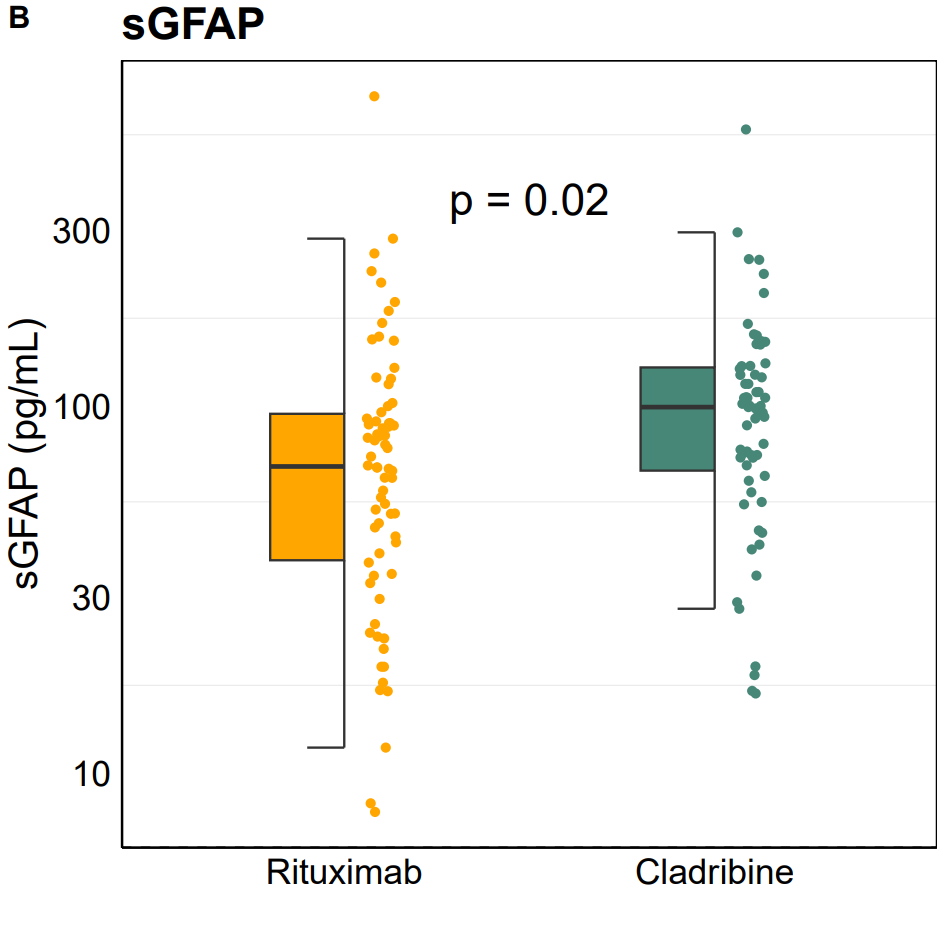


sNfL and sGFAP were available for analysis in 133 patients (47%) from samples collected 1.7 to 4.2 years after initiation of rituximab or cladribine.

1. sNfL of 133 patients with MS (cladribine, n = 63; rituximab, n =70). The levels of log(sNfL) were compared between the groups using linear regression adjusting for baseline covariates.
2. sGFAP of 133 patients with MS (cladribine, n = 63; rituximab, n =70). The levels of log(sGFAP) were compared between the groups using linear-regression analysis adjusting for baseline covariates. The geometric mean of sGFAP in cladribine-treated patients was 87.8 pg/mL and 62.6 pg/mL in the rituximab-treated patients.

The baseline covariates included in the logistic regression analysis were age; sex; disease duration; number of previous DMTs; number of T2-lesions on MRI; EDSS score; relapses within 12 months prior to baseline; MRI lesion activity within 12 months prior to baseline; time from baseline MRI to baseline; and reasons for discontinuing the last DMT prior to the index therapy.

**Abbreviations**: sNfL, serum neurofilament light chain; sGFAP, serum glial fibrillary acidic protein. MRI, magnetic resonance imaging; DMT, disease-modifying therapy; EDSS, the Expanded Disability Status Scale.

eFigure 4. Cumulative incidence of new MRI disease activity in subgroups.


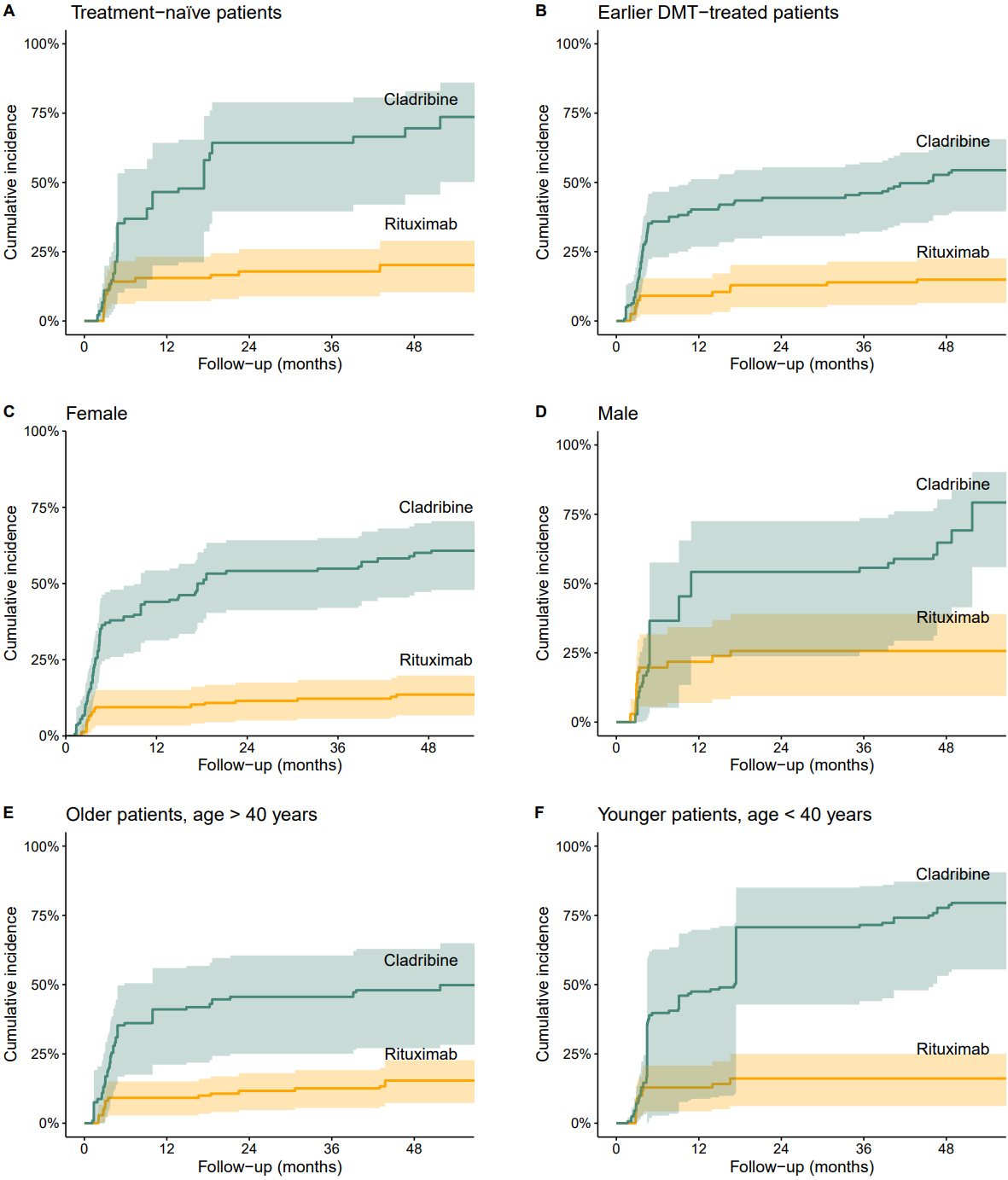


Cumulative incidence of new MRI disease activity, defined as new T2-lesions on brain or spinal cord MRI, compared to baseline MRI up to 5 years after index treatment initiation. Shaded areas represent 95% confidence intervals.

Adjustments for baseline covariates (age; sex; disease duration; number of previous DMTs; number of T2-lesions on MRI; EDSS score; relapses within 12 months prior to baseline; MRI lesion activity within 12 months prior to baseline; time from baseline MRI to baseline; and reasons for discontinuing the last DMT prior to the index therapy) were performed using stabilized inverse probability of treatment (propensity score) weights, estimated using logistic regression.

**Abbreviations**: MRI, magnetic resonance imaging; DMT, disease-modifying therapy; EDSS, the Expanded Disability Status Scale.

eFigure 5. Cumulative incidence of new MRI disease activity from re-baseline MRI.


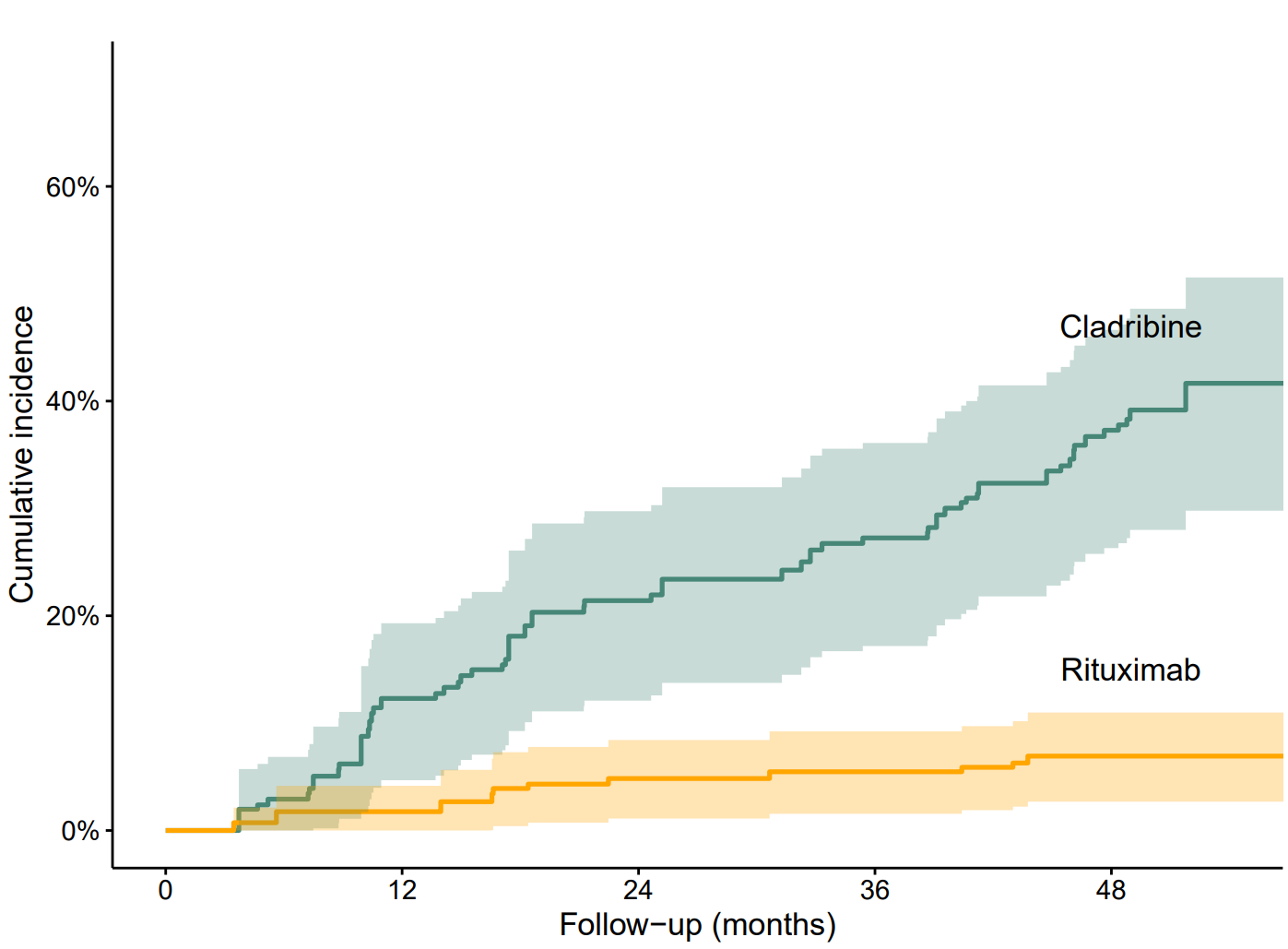


Baseline (x = 0) reflects the timepoint when re-baseline MRI was conducted.

Cumulative incidence of new MRI disease activity, defined as new T2-lesions on brain or medullary MRI, up to 5 years after the re-baseline MRI (the first new MRI after baseline MRI). The rebaseline MRIs were conducted a median of 3.6 months after initiation of cladribine and 3.0 months after initiation of rituximab. Shaded areas represent 95% confidence intervals.

Adjustment for baseline covariates (age; sex; disease duration; number of previous DMTs; number of T2-lesions on MRI; EDSS score; relapses within 12 months prior to baseline; MRI lesion activity within 12 months prior to baseline; time from baseline MRI to treatment initation; and reasons for discontinuing the last DMT prior to the index therapy) was performed using stabilized inverse probability of treatment (propensity score) weights, estimated using logistic regression.

**Abbreviations**: MRI, magnetic resonance imagin; DMT, disease-modifying therapy; EDSS, the Expanded Disability Status Scale.

eFigure 6. Cumulative incidence of new MRI disease activity with baseline set to 18 months after treatment initiation


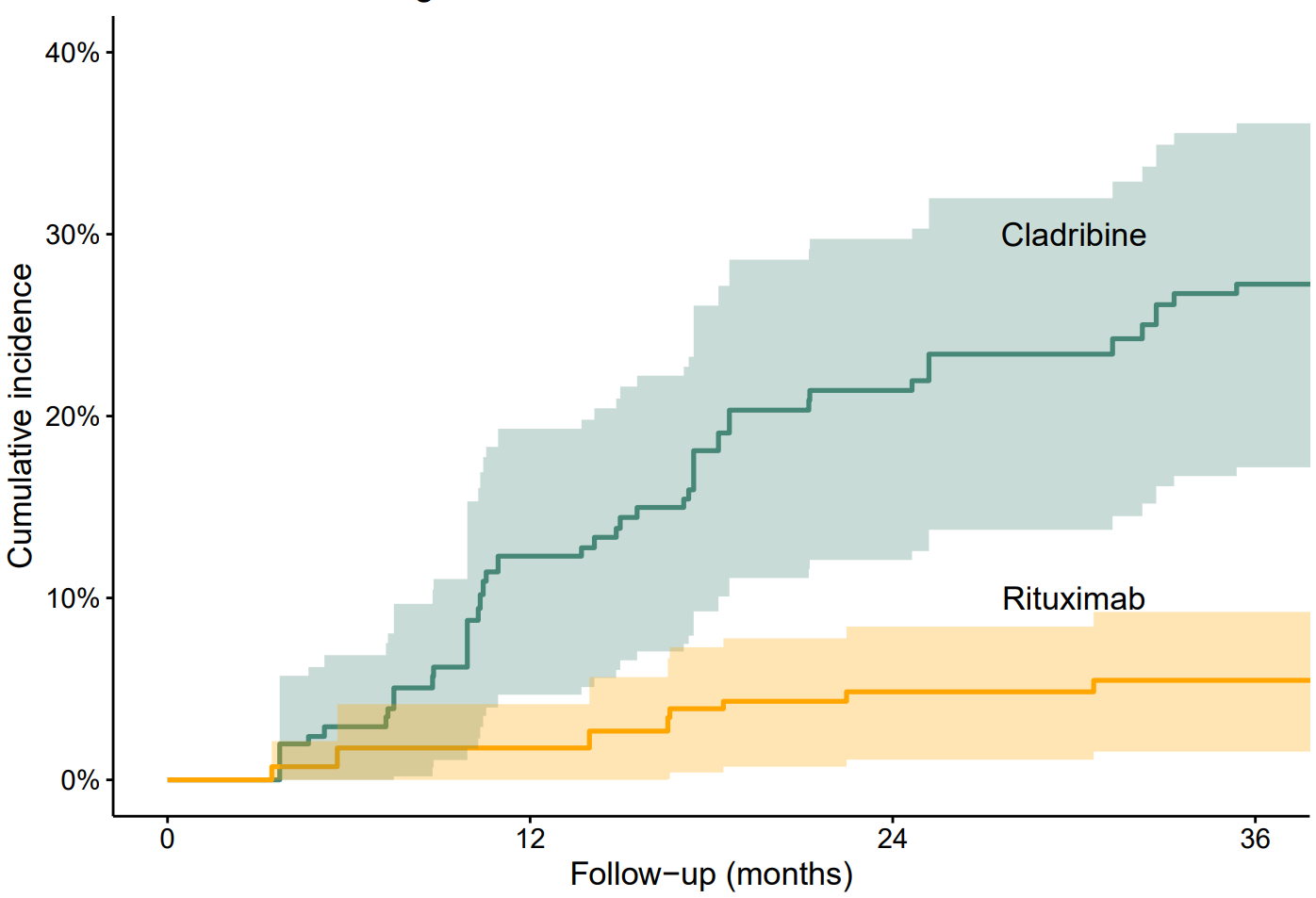


Baseline (x = 0) reflects 18 months after treatment initiation.

Cumulative incidence of new MRI disease activity, defined as new T2-lesions on brain or medullary MRI, with baseline set to 18 months after treatment initiation and 3 years follow-up.

Patients who switched from their index therapy before 18 months or had no follow-up MRI after 18 months were excluded. Shaded areas represent 95% confidence intervals.

Adjustment for baseline covariates (age; sex; disease duration; number of previous disease modifying therapies (DMTs); number of T2-lesions on MRI; EDSS score; relapses within 12 months prior to baseline; MRI lesion activity within 12 months prior to baseline; time from baseline MRI to treatment initation; and reasons for discontinuing the last DMT prior to the index therapy) was performed using stabilized inverse probability of treatment (propensity score) weights, estimated using logistic regression

**Abbreviations**: MRI, magnetic resonance imaging; DMT, disease-modifying therapy; EDSS, the Expanded Disability Status Scale.

eFigure 7. Cumulative incidence of new MRI disease activity by treatment strategy cohorts


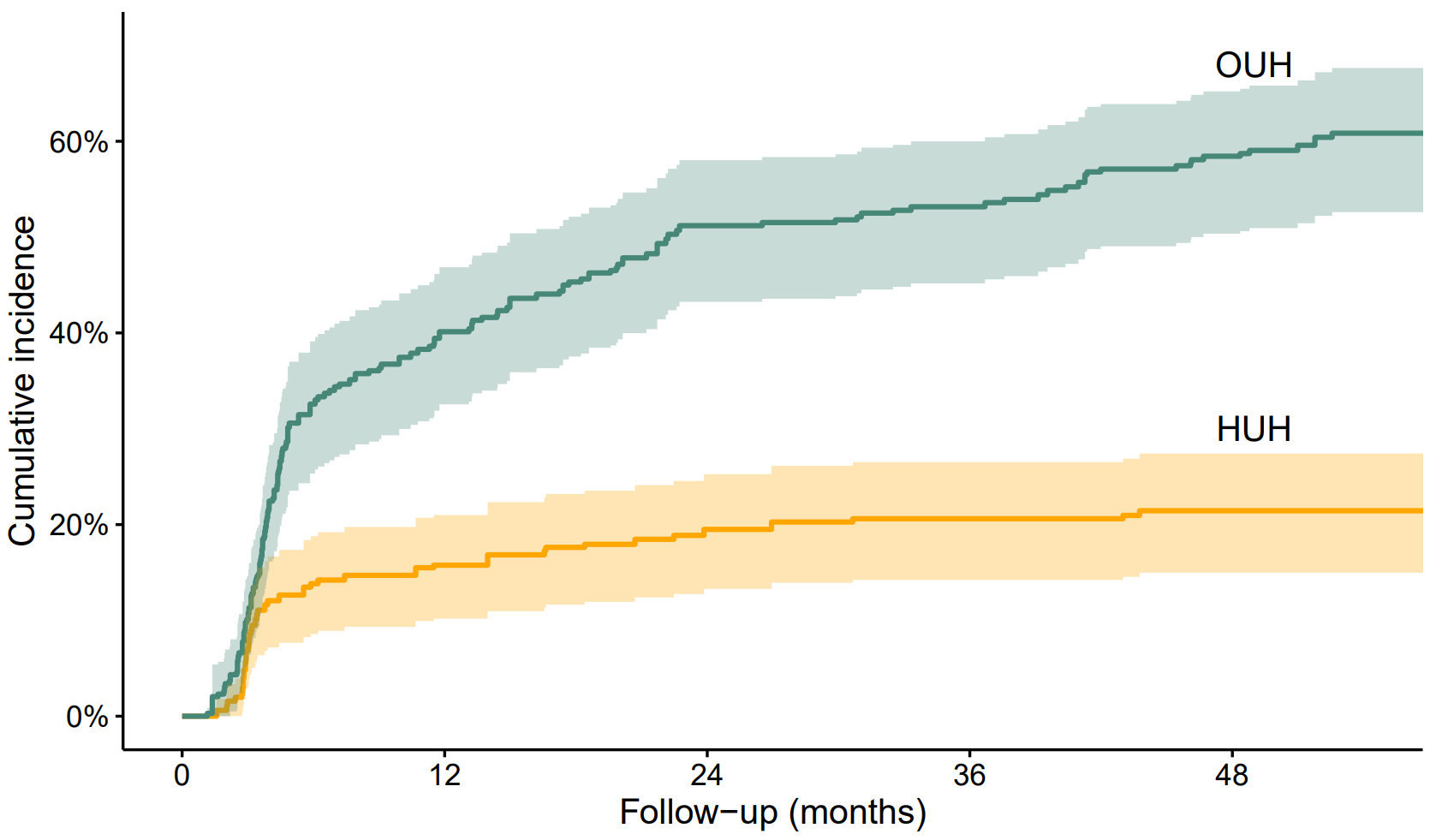


Cumulative incidence of new MRI disease activity, defined as new T2-lesions on brain or medullary MRI, up to 4.5 years after treatment initiation at the two centers. At Oslo university hospital (OUH), 49% of patients with relapsing-remitting multiple sclerosis were initiated on cladribine, while at Haukeland university hospital (HUH), 80% of patients were initiated on rituximab.

Adjustment for baseline covariates (age; sex; disease duration; number of previous DMTs; number of T2-lesions on MRI; EDSS score; relapses within 12 months prior to baseline; MRI lesion activity within 12 months prior to baseline; time from baseline MRI to baseline; and reasons for discontinuing the last DMT prior to the index therapy) was performed using stabilized inverse probability of treatment (propensity score) weights, estimated using logistic regression.

**Abbreviations**: MRI, magnetic resonance imaging; DMT, disease-modifying therapy; EDSS, the Expanded Disability Status Scale; OUH, Oslo university hospital; HUH, Haukeland university hospital.

eFigure 8. Cumulative incidence of new MRI disease activity by treatment, independent of hospital


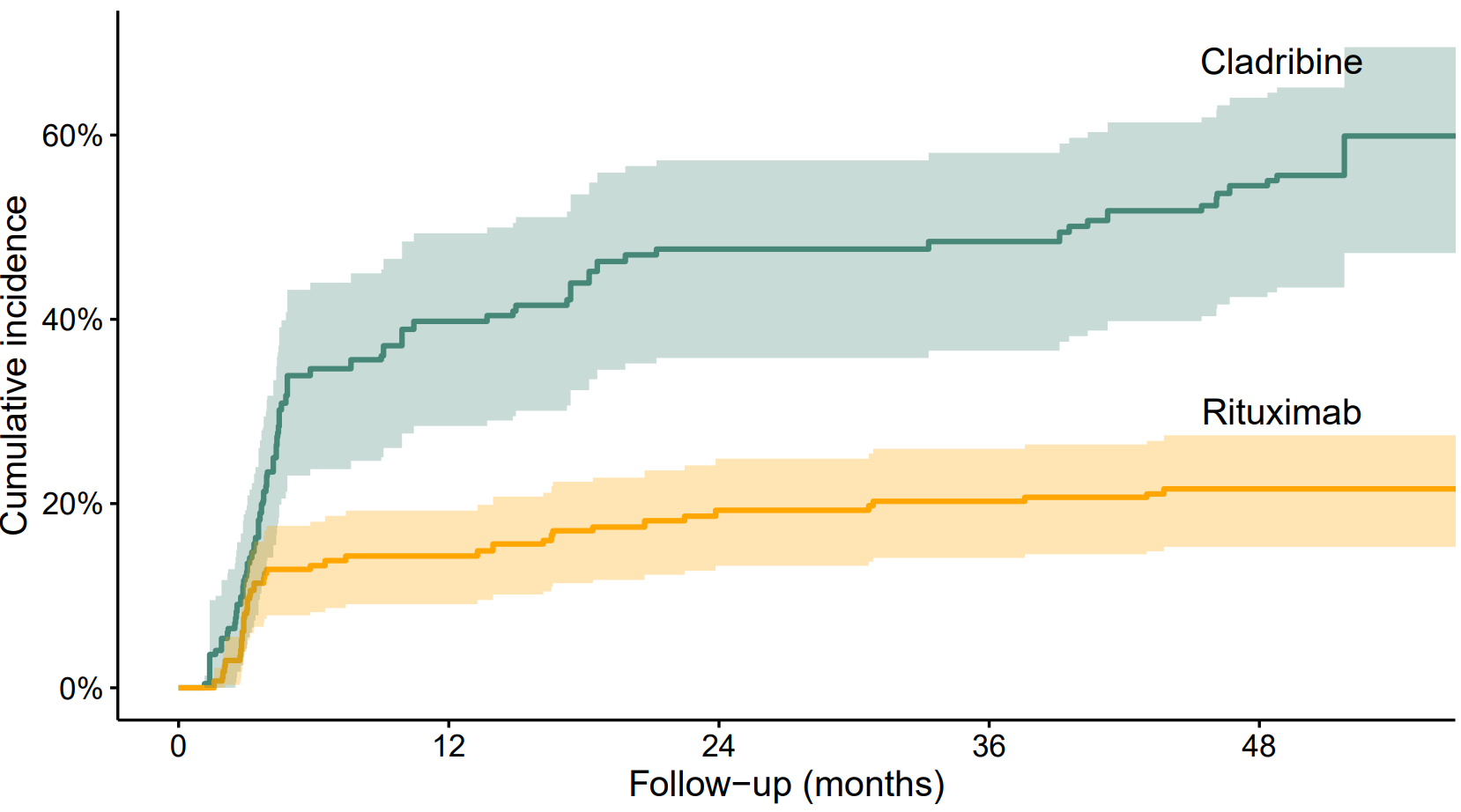


Cumulative incidence of new MRI disease activity, defined as new T2-lesions on brain or medullary MRI, up to 4.5 years after baseline by treatment independent of hospital.

Adjustment for baseline covariates (age; sex; disease duration; number of previous disease modifying therapies (DMTs); number of T2-lesions on MRI; EDSS score; relapses within 12 months prior to baseline; MRI lesion activity within 12 months prior to baseline; time from baseline MRI to baseline; and reasons for discontinuing the last DMT prior to the index therapy) was performed using stabilized inverse probability of treatment (propensity score) weights, estimated using logistic regression.

**Abbreviations**: MRI, magnetic resonance imaging; DMT, disease-modifying therapy; EDSS, the Expanded Disability Status Scale.

| eTable 1**. Specification and emulation of the target trial** | | |
| --- | --- | --- |
| **Protocol component** | **Target trial specification** | **Target trial emulation** |
| Eligibility criteria | - Aged ≥18 years old between May 15, 2018, and October 15, 2019. - Diagnosis of relapsing-remitting MS according to the 2017 McDonald criteria. - Disease activity, defined as relapse or MRI activity during the last 12 months. - No previous use of rituximab or cladribine. | Same as for the target trial, except:   - Disease activity the year prior to baseline is not a criterion, but an MRI conducted within 12 months prior to baseline, or up to 1 month following, is. - At least 1 MRI conducted after baseline within follow-up. |
| Treatment strategies | 1. Rituximab as infusions at baseline (1000 mg), then every 6 months (500 mg). 2. Cladribine as tablets given at baseline and after 1 year (a total of 3.5 mg/kg). | Same as for target trial, but *initiating* therapy is the exposure:   1. At least one dose of rituximab 2. At least one dose of cladribine |
| Treatment assignment | Randomization rituximab:cladribine is 1:1.  The radiologists, assessing the primary endpoint, are blinded.  Individuals and clinicians are aware of the assigned treatment strategy. | We assume random assignment for individuals who received rituximab to those who received cladribine after weighting for baseline covariates that might be prognostically important. |
| Outcomes | Primary: Time to first new MRI T2 lesion from MRI conducted at baseline  Secondary:   - Time to first relapse from baseline - Time to treatment discontinuation from baseline - Time to confirmed disability progression and confirmed disability improvement - Number of new or enlarging cerebral MRI T2 lesions 4 years after baseline - Change of sNfL and sGFAP from baseline to 2 and 4 years after baseline. | Primary: Same as for the target trial, except that the baseline MRI is considered the most recent MRI conducted within the year prior to initiation of therapy, or up to 1 month following, as not all patients necessarily had MRIs conducted exactly at baseline.  Secondary: Same, but number of new or enlarging cerebral MRI T2 lesions cannot be evaluated, as the exact numbers of lesions or expanding lesions are not available in the NMSRB. Since consecutive serum samples are not available, point measurements of sNfL and sGFAP will be compared. |
| Follow-up | Follow-up starts on the day of the first dose of index therapy (baseline) and ends on the day of the outcome of interest, death, or 4 years after treatment initiation, whichever happens first. | Same as for target trial, except not ending 4 years after treatment initiation but at the end of the study period (August 31, 2023). |
| Causal contrasts | Intention-to-treat effect and  Per-protocol effect | Observational analogue of the intention-to-treat effect. |
| Statistical analysis | Cumulative incidence (risk) curves and estimates of the 4-year risk, risk differences and risk ratios.  Subgroup analyses by age, sex and treatment history (treatment naïve or not). Sensitivity analysis comparing the treatment strategies, including all patients starting on any DMT at HUH and OUH. | Same as for the target trial. Though, in addition, we conduct a sensitivity analysis on time to new MRI disease activity from the “re-baseline” MRI. |
| **Abbreviations**: MS, multiple sclerosis; MRI; magnetic resonance imaging; EDSS, the Expanded Disability Status Scale; sNfL, serum neurofilament light chain; sGFAP, serum glial fibrillary acidic protein; NMSRB, The Norwegian MS Registry and Biobank; DMT disease-modifying therapy; HUH, Haukeland university hospital; OUH, Oslo university hospital. | | |

| eTable 2. **Comparative effectiveness 6 months after initiation of rituximab (n= 159) and cladribine (n = 126) in patients with multiple sclerosis.** | | | | | | |
| --- | --- | --- | --- | --- | --- | --- |
| Outcome | **Number of events** | | **6-month risk (95% CI)** | | **Risk difference^c^**  **(95% CI)** | **Risk ratio^c^**  **(95% CI)** |
|  | Rituximab | Cladribine | Rituximab | Cladribine |  |  |
|  |  |  |  |  | *percentage-points* |  |
| New MRI activity^a^ | 19 | 46 | 12%  (6.4 to 17) | 35%  (24 to 44) | 23.4  (14.7 to 34.9) | 0.34  (0.19 to 0.51) |
| New relapse | 3 | 11 | 1.8%  (0 to 3.8) | 7.0%  (2.6 to 11) | 5.2  (1.7 to 12.4) | 0.26  (0.00 to 0.68) |
| Treatment discontinuation ^b^ | 0 | 0 | 0%  (0 to 0) | 0%  (0 to 0) | 0  (0 to 0) | NaN |
| ^a^ New MRI disease activity, defined as new T2-lesions on brain or spinal cord MRI compared to baseline MRI.  ^b^ Treatment discontinuation or third dose of cladribine, among cladribine-treated patients.  ^c^ Adjusted for age; sex; disease duration; number of previous disease-modifying therapies (DMTs); number of T2-lesions on MRI; EDSS score; relapses within 12 months prior to baseline; MRI lesion activity within 12 months prior to baseline; time from baseline MRI to baseline; and reasons for discontinuing the last DMT prior to the index therapy.  Abbreviations: CI, confidence interval; MRI, magnetic resonance imaging. | | | | | | |

| eTable 3. **Comparative effectiveness 2 years after initiation of rituximab (n= 159) and cladribine (n = 126) in patients with multiple sclerosis.** | | | | | | |
| --- | --- | --- | --- | --- | --- | --- |
| Outcome | **Number of events** | | **2-year risk (95% CI)** | | **Risk difference^c^**  **(95% CI)** | **Risk ratio^c^**  **(95% CI)** |
|  | Rituximab | Cladribine | Rituximab | Cladribine |  |  |
|  |  |  |  | | *percentage-points* |  |
| New MRI activity^a^ | 25 | 61 | 15%  (9.3 to 21) | 49%  (37 to 59) | 33.8  (22.8 to 43.2) | 0.31  (0.21 to 0.47) |
| New relapse | 4 | 16 | 2.7%  (0 to 5.4) | 13%  (5.7 to 20) | 10.4  (4.3 to 16.4) | 0.21  (0.04 to 0.49) |
| Treatment discontinuation ^b^ | 3 | 9 | 2.7%  (0 to 6.0) | 7.9%  (2.1 to 13) | 5.1  (0.2 to 10.3) | 0.35  (0.00 to 0.86) |
| ^a^ New MRI disease activity, defined as new T2-lesions on brain or spinal cord MRI compared to baseline MRI.  ^b^ Treatment discontinuation or third dose of cladribine, among cladribine-treated patients.  ^c^ Adjusted for age; sex; disease duration; number of previous disease-modifying therapies (DMTs); number of T2-lesions on MRI; EDSS score; relapses within 12 months prior to baseline; MRI lesion activity within 12 months prior to baseline; time from baseline MRI to baseline; and reasons for discontinuing the last DMT prior to the index therapy.  Abbreviations: CI, confidence interval; MRI, magnetic resonance imaging. | | | | | | |

| eTable 4. Reason for discontinuation or third dose of cladribine | | |
| --- | --- | --- |
| **Reason for discontinuation** | **No. (%)** | |
|  | **Rituximab**  N = 11 | **Cladribine**  N = 35 |
|  |  |  |
| Disease activity | 2 (18) | 24 (69) |
| Adverse events | 6 (55) | 1 (3) |
| Other reasons^a^ | 3 (27) | 2 (6) |
| Unknown | 0 (0) | 8 (23) |

^a^include family planning, patient’s decision and other reasons.

| eTable 5. Timing of adverse events after treatment initiation | | |
| --- | --- | --- |
|  | **Hospitalizations related to adverse events, per 100 patient-years**  (Number of hospitalizations / patient years of observation on DMT monotherapy) | |
|  | **Rituximab**  **N = 158** | **Cladribine**  **N = 126** |
| Year 1 | **6.4**  (10/157.3) | **2.4**  (3/125.5) |
| Year 2 | **2.6**  (4/155.8) | **5.0**  (6/119.5) |
| Year 3 | **1.3**  (2/154.1) | **5.2**  (6/116.3) |
| Year 4^a^ | **11.3**  (17/149.8) | **4.6**  (5/109.7) |
| Year 5^b^ | **11.7**  (9/76.7) | **2.3**  (1/44.4) |
| Year 6 | **0**  (0/2.8) | **0**  (0/0.4) |
| **Total** | **6.0**  (42/696.5) | **4.1**  (21/515.4) |

^a^ Corresponding to 2021-2022. ^b^ Corresponding to 2022-2023.

**Abbreviations**: DMT, disease-modifying therapy.

| eTable 6. Baseline variables by treatment strategy cohort (unweighted) | | |
| --- | --- | --- |
| **Characteristic** | **HUH**  N = 193 | **OUH**  N = 243 |
| Therapy started as first in baseline-period |  |  |
| Alemtuzumab | 5 (3%) | 19 (8%) |
| Cladribine | 1 (1%) | 119 (49%) |
| Dimethyl fumarate | 2 (1%) | 5 (2%) |
| Fingolimod | 3 (2%) | 18 (7%) |
| Glatiramer acetate | 2 (1%) | 10 (4%) |
| HSCT | 4 (2%) | 2 (1%) |
| Natalizumab | 2 (1%) | 0 (0%) |
| Rituximab | 155 (80%) | 26 (11%) |
| Teriflunomide | 19 (9%) | 44 (18%) |
| Age, mean (SD), y | 42 (11) | 40 (11) |
| Sex |  |  |
| Female | 143 (74%) | 179 (74%) |
| Male | 50 (26%) | 64 (26%) |
| Disease duration, median (IQR), y | 5 (11) | 6 (12) |
| No. of previous DMTs |  |  |
| 0 | 95 (49%) | 109 (45%) |
| 1 | 35 (18%) | 60 (25%) |
| 2 | 35 (18%) | 37 (15%) |
| 3 | 18 (9%) | 20 (8%) |
| 4 - 6 | 10 (5%) | 17 (7%) |
| MRI, count of T2 lesions |  |  |
| 0 - 5 | 24 (12%) | 56 (23%) |
| 6 - 10 | 52 (27%) | 31 (13%) |
| >10 | 117 (61%) | 156 (64%) |
| Unknown | 0 (0%) | 0 (0%) |
| Disability, EDSS score^a^ |  |  |
| Mild, 0 – 2 | 50 (26%) | 57 (23%) |
| Moderate, 2.5 – 5 | 19 (10%) | 23 (10%) |
| Severe, 5.5 and higher | 5 (3%) | 6 (3%) |
| Unknown | 119 (62%) | 157 (65%) |
| Relapses within 12 months before baseline | 107 (55%) | 99 (41%) |
| MRI activity within 12 months before baseline | 134 (69%) | 191 (79%) |
| Reason for discontinuing the DMT before baseline |  |  |
| No DMTs prior to index treatment | 95 (49%) | 109 (45%) |
| Other reasons^b^ | 21 (11%) | 49 (20%) |
| Side effects | 28 (15%) | 44 (18%) |
| Treatment failure | 47 (24%) | 35 (14%) |
| Unknown | 2 (1%) | 6 (3%) |
| Time between the baseline MRI and index treatment start, median (IQR), d | 28 (54) | 60 (78) |

^a^ The last reported EDSS score within the year prior baseline. ^b^ Include family planning, patient’s decision and other reasons. **Abbreviations**: HUH, Haukeland university hospital; OUH, Oslo university hospital; DMTs, disease-modifying therapies; y, years; MRI, magnetic resonance imaging; EDSS, the Expanded Disability Status Scale; MS, multiple sclerosis.
